# Pectin and Neutral Monosaccharides Production during the Simultaneous Hydrothermal Extraction of Waste Biomass from Refining of Sugar—Optimization with the Use of Doehlert Design

**DOI:** 10.3390/molecules24030472

**Published:** 2019-01-29

**Authors:** Hanna Pińkowska, Małgorzata Krzywonos, Paweł Wolak, Adrianna Złocińska

**Affiliations:** 1 Wrocław University of Economics, Department of Industrial Chemistry, Komandorska 118/120, 53-345 Wrocław, Poland; hanna.pinkowska@ue.wroc.pl (H.P.); pawel.wolak@ue.wroc.pl (P.W.); 2 Wrocław University of Economics, Department of Bioprocess Engineering, Komandorska 118/120, 53-345 Wrocław, Poland; 3 Wrocław Medical University, Laboratory of Elemental Analysis and Structural Research, Borowska 211A, 50-556 Wrocław, Poland; adrianna.zlocinska@umed.wroc.pl

**Keywords:** sugar beet pulp, hydrothermal extraction, hydrothermolysis, pectin, neutral monosaccharides, experimental design methodology

## Abstract

We propose a one-stage hydrothermal extraction of sugar beet pulp leading to effective co-production of pectin and neutral monosaccharides with a relatively high yield and satisfactory purity without the presence of an acidic catalyst. The optimal experimental design methodology was used for modelling and optimizing the yield of pectin and neutral monosaccharides. In good agreement with experimental results (R^2^ = 0.955), the model predicts an optimal yield of pectin (approx. 121.1 g kg^−1^ ± 0.47 g kg^−1^) at a temperature and time of about 118.1 °C and 21.5 min, respectively. The highest yield of the sum of neutral monosaccharides (approx. 82.6 g kg^−1^ ± 0.72 g kg^−1^) was obtained at about 116.2 °C and 26.4 min (R^2^ = 0.976). The obtained results are suitable for industrial upscaling and may provide an incentive to implement a new, environmentally friendly, simple, and effective method for treating waste product from the sugar refining industry, which has proved onerous until now.

## 1. Introduction

Pectin is a valuable product with attractive functional properties [1] and an annual growth rate of approx. 5% [2], which is widely approved for use in foods as a safe additive [1,2,3].

Sugar beet (*Beta vulgaris* L.) pulp (SBP) is a pectin-rich (15–30% dry weight) [3] byproduct of the sugar refining industry and traditionally commonly used as a raw material for livestock feeds. Only a few attempts have been made to utilize excess SBP for other purposes, such as the production of pectin [4,5,6,7], some using hydrothermal conditions [8,9].

In SBP, also a high content of a lignocellulose fraction (hemicellulose up to 30% dry weight, cellulose up to 24% dry weight, and small amounts of lignin) has been determined [1]. After hydrothermal pectin extraction and partial conversion to neutral monosaccharides, SBP may also be a relevant raw material for the production of biofuels and fine chemicals such as furfurals, liquid aliphatic hydrocarbons, ethanol, acids, and diols [2,3]. Since hydrothermal technologies do not require the addition and recovery of chemicals except for water, in consequence, they allow valuable materials to be produced with zero emissions [3]. Therefore, hydrothermal processes are regarded as environmentally friendly, cost-effective, and possessing great potential for practical applications [10,11,12,13].

In this work, the valuable properties of subcritical water were exploited in the reuse of components contained in SBP according to the concept of biorefineries, which implies the conversion of the dominant polysaccharide fraction of SBP into useful semi-finished bioproducts. The concept assumes the use of hydrothermal extraction of pectin and simultaneous production of neutral monosaccharides formed as a result of partial hydrothermolysis of the polysaccharide components of SBP. In the present research, the effect of the extraction parameters (temperature (*T*) and time (*t*)) on the yield of pectin and neutral monosaccharides was determined. The optimal experimental design (OED) methodology was used to establish a statistically significant extraction models and optimize the conditions for both pectin extraction and neutral monosaccharides production.

## 2. Results and Discussion

### 2.1. Composition of Sugar Beet Pulp

The chemical composition of SBP is shown in Table 1. 

This composition is in agreement with previously published data [4]. The obtained values for degree of methylation (DM) and degree of acetylation (DA) were 42.5% and 56.0% mol, respectively, which are characteristic features of SBP [5]. The density of SBP is 0.596 g mL^−1^, and the pH of 100 g L^−1^ water slurry has a value of 5.14.

### 2.2. Modelling of Hydrothermal Extraction of Pectin from SBP Using Optimal Experimental Design

In Table 2 are listed the temperature and time of the conducted experiments and the pectin yield values (*Y_P_*) of the experimental and calculated responses. 

For best results concerning *Y_P_*, the extraction temperature and time should not exceed 120 °C and 15 min, or 110 °C and 30 min. The highest *Y_P_*, 118.0 g kg^−1^ (average experimental values), was obtained at the center of the experimental region (Experiments 7–7’’). According to the obtained experimental results, the coefficients of the quadratic polynomial model were calculated (Equation (1)):

*Y_P_* = 111.8 + 3.4 *x*_1_ + 18.5 *x*_2_ − 28.4 *x*_1_^2^ − 28.7 *x*_2_^2^ − 21.9 *x*_1_*x*_2_ (1)

Standard deviations of the coefficients are given in Table S1A (Supplementary Materials).

**Figure 1:**
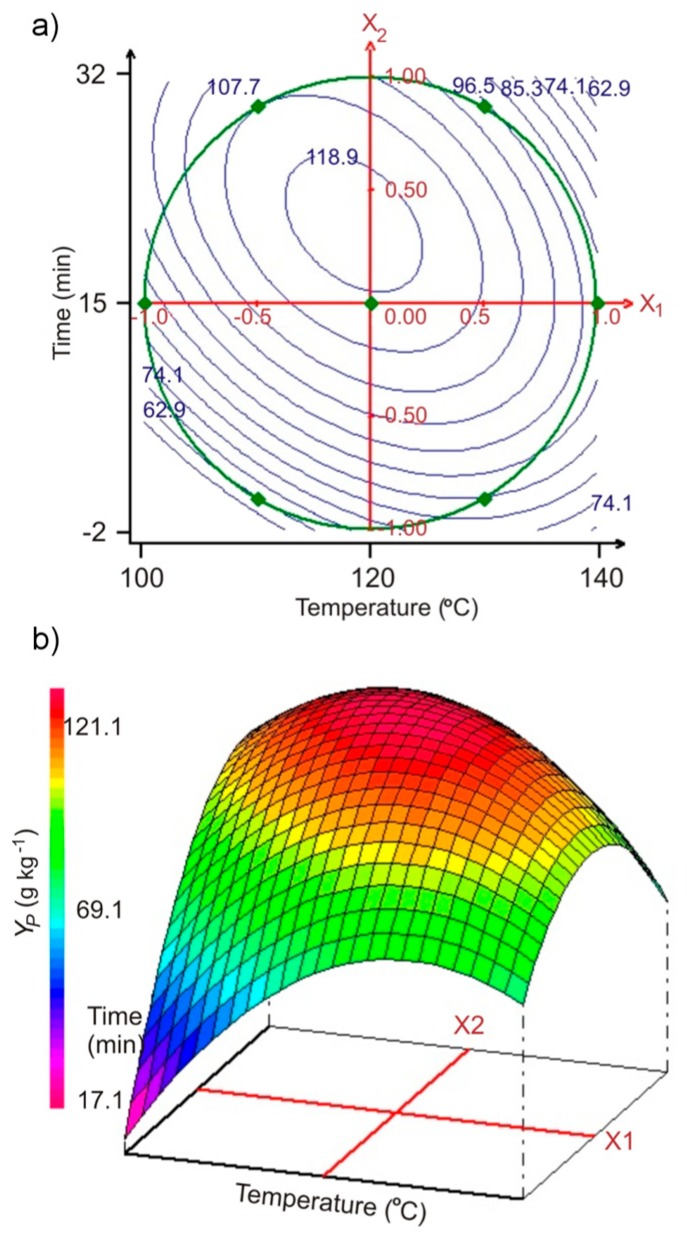
(**a**) Contour plots of pectin yield Y_P_ versus extraction temperature and time of SBP hydrothermal treatment (representations based on the quadratic polynomial model, Equation (1); results obtained from Doehlert matrix, Table 2); (**b**) corresponding three-dimensional plot.

The predicted contour plot and the three-dimensional representation of the same plot are shown in Figure 1a,b, respectively. The graphic analysis showed that the hydrothermal extraction of pectin proceeded most efficiently at temperatures of 112.6 to 124.9 °C and times of 26.2 to 15.8 min, respectively, with a predicted optimum *Y_P_* value of 121.1 g kg^−1^ (± 0.47) at 118.1 °C and 21.5 min, in good agreement with experimental results (R^2^ = 0.955). The experimental validation data are described in Supplementary Materials (Tables S2A–S5A).

In the performed experimental series, *Y_P_* values were lower than those obtained both by conventional acid extraction [6,7,8] and by ultrasonic-assisted treatment combined with subcritical water [9,10], since pectin produced by hydrothermal extraction was subsequently hydrolyzed to degraded products in the second step of process [11,12,13,14,15]. The first stage of the degradation was interpreted as the hydrolysis of the weakest bonds in rhamnogalacturonan regions (between rhamnose and galacturonic acid units), and the second as a hydrolysis of the strongest bonds between galacturonic acids units in the main chain of pectin [14,15], leading to the formation of primary hydrothermolysis products (uronic acids, acetic acid), monosaccharides (pentoses and hexoses), secondary hydrothermolysis products (carboxylic acids and furfurals) (Table S6A, Supplementary Materials), and also degradation and gasification products unidentified in this work. 

### 2.3. Characterization and Chemical Composition of Pectin

**Figure 2:**
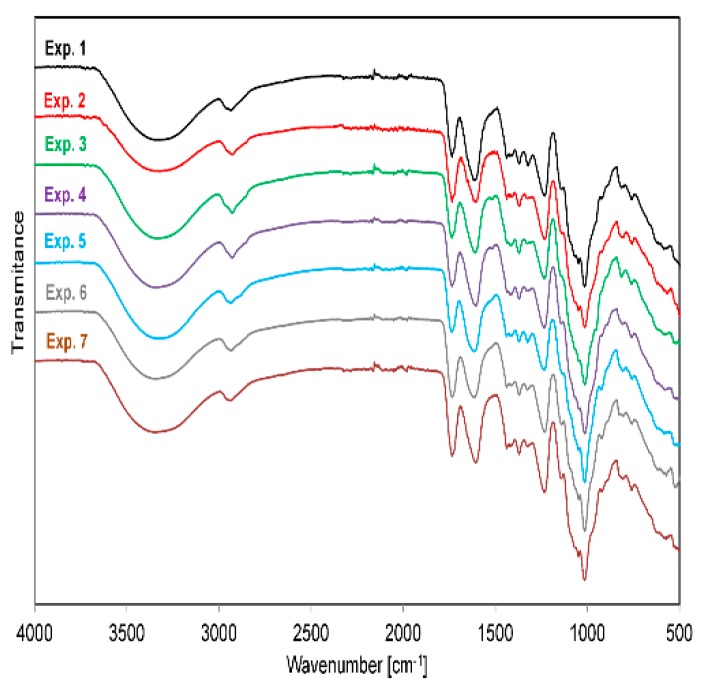
FTIR spectra of pectin samples in Experiments 1–7.

The ATR-FTIR spectra of pectin samples obtained in the experimental series (Experiments 1–7) in the range of 4000–800 cm^−1^ are shown in Figure 2. 

The spectra present the typical chemical groups of pectin [1,6,16,17,18,19,20,21]. The broad, strong band observed in the range 3670–3000 cm^−1^ (3346 cm^−1^) refers to O-H stretching absorption due to inter- and intramolecular hydrogen bonds of galacturonic acid. The sharp, double overlapping stretching bands around 3000–2763 cm^−1^ (2926 cm^−1^) were assigned to C-H absorption [16]. These include pectin CH, CH_2_, and CH_3_ stretching and bending vibrations, due to the methyl esters of galacturonic acid [1]. Two moderately intense bands were observed in the C-H region of aliphatic compounds. The C-H stretching and bending vibrations are seen as a band superimposed upon the broader O-H band, which is typically observed with pectin. Due to a large O-H stretching response occurring in a broad region at 3670–2500 cm^−1^, the O-CH_3_ stretching band is masked and is not a reliable indicator of pectin degree of methoxylation. In contrast, strong bands occurring at 1820–1690 cm^−1^ (1735 cm^−1^) and 1640–1600 cm^−1^ (1605 cm^−1^) indicate the ester carbonyl (C=O) groups and carboxylate ion asymmetrical stretching band (COO-), respectively [16]. The intensity difference of these peaks suggests that it could be a low methoxyl pectin [1]. The bands at 1560–1540 cm^−1^ and 1500–1600 cm^−1^, assigned to the presence of protein and aromatic ring stretching, respectively [17], were not observed on the spectrum, which suggests that protein and lignin were absent in the pectin-enriched product. Carboxylate groups also show a weaker symmetric stretching band near 1400 cm^−1^. Other bands of lesser importance in pectin are weak bands of C-H bending, occurring at 1370 cm^−1^, and C–O stretching occurring at 1300–930 cm^−1^ [17,21]. Structural features arising from particular conformations around the glycosidic bonds of pectin were observable in the 1100–930 cm^−1^ range. The band at 1142 corresponds to the ring vibration and C-OH bending, while the prominent band at 1015 cm^−1^ is attributable to the C-OH bending [6]. However, according to Gnanasambandam and Proctor [16], even a small difference in pectin source, applied extraction technique, or used parameters, and also differences in the structure and composition of pectin can result in both small and significant changes in the absorption peaks.

In all experiments, we obtained two fractions: the first contained high molecular weight pectin (more than 642 kDa) and the second consisted of pectin with particles of small size (less than 6 kDa). The obtained results were close to those presented in a study by Yapo et al. [7]. 

The chemical composition of pectin is shown in Table 3. In the pectin extracted under optimal conditions, the uronic acids content was 512.8 g kg^−1^, total monosaccharides 310.7 g kg^−1^ (arabinose 122.8 g kg^−1^, glucose 9.7 g kg^−1^, xylose 12.8 g kg^−1^, and the sum of galactose, mannose, and rhamnose 165.4 g kg^−1^), DM 51.9 % mol [7], and DA 16.7 % mol [7]. 

The amounts of uronic acids in all pectin extracts were close to the results obtained in hydrothermal conditions by Ref. [9], but they were only moderately influenced by extraction temperature and time [7], and were lower than the data already published Ref. [8,22]. According to Yapo et al. [7], the extraction yield was not related to the content in uronic acids. In contrast, the main parameter influencing the uronic acids content in pectin extracts was the pH of the extraction mixture: with decreasing pH, the uronic acids content increased. In this study, the initial pH of extraction mixtures had a relatively high value of 5.14 and, for this reason, all the obtained pectin extracts were not sufficiently pure.

The content of monosaccharides in the pectin obtained at the higher extraction temperature and longer time was higher than the values obtained at lower temperature and shorter time. This indicates that in harsher extraction conditions, the hydrothermolysis of pectin occurred [15]. The main monosaccharides present in the pectin extracts were arabinose and the sum of galactose, mannose, and rhamnose, and their content was close to the data already published [7,10]. Other neutral monosaccharides, such as xylose and glucose, were also present but in lower amounts, and were therefore assumed to be contaminants from the partial hydrothermal decomposition of hemicellulose.

In comparison with literature data, the DM in pectin was in good agreement with values reported in previous studies, but the DA was slightly lower than in pectin extracted with mineral acid in optimal extraction conditions [5,7,23], especially in the case of pectin obtained at a higher extraction temperature (130 and 140 °C), because harsher conditions of hydrothermal extraction additionally increased the deacetylation of the polygalacturonic chain [7].

Many factors affect the structure and morphology of pectin, such as structure and chemical composition of raw material rich in pectin, the manner and conditions of its storage (temperature, time, and atmosphere) [19,21], as well as the method and product extraction and the process parameters used [24]. 

Pectin obtained in our study via one-stage hydrothermal extraction of SBP, as well as those obtained by Chen et al. [9] in ultrasonic-assisted treatment combined with subcritical water and via traditional extraction processes in aqueous acid medium, are characterized by low molecular weight, high content of neutral monosaccharides, and low to average values of DM and DA [7,25]. 

### 2.4. Modelling of Hydrothermolysis of Polysaccharide Components of SBP, Using Optimal Experimental Design

Table 2 lists the values of the experimental and calculated responses of the sum of neutral monosaccharides (*Y_NS_*). The *Y_NS_* can be predicted by Equation (2):

*Y_NS_* = 78.4 + 3.7 *x*_1_ + 14.2 *x*_2_ − 32.8 *x*_1_^2^ − 13.9 *x*_2_^2^ − 22.4 *x*_1_*x*_2_ (2)

Standard deviations of the coefficients are given in Table S1A (Supplementary Materials).

**Figure 3:**
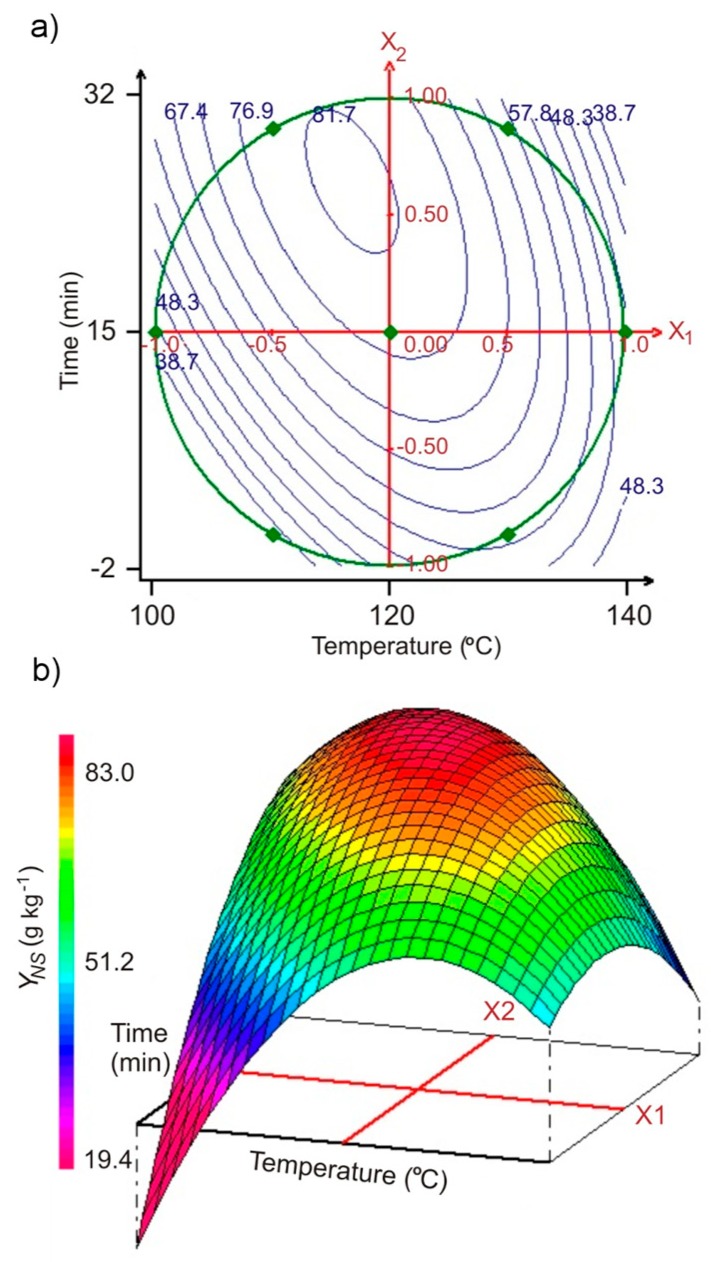
(**a**) Contour plots of the sum of neutral monosaccharides yield *Y_NS_* versus hydrolysis temperature and time of SBP hydrothermal treatment (representations based on the quadratic polynomial model, Equation (2); results obtained from Doehlert matrix, Table 2); (**b**) corresponding three-dimensional plot.

From the above model were drawn two- (Figure 3a) and three-dimensional (Figure 3b) representations of the *Y_NS_* as a function of SBP hydrothermal treatment temperature and time. In the performed experimental series, the highest yield of the *Y_NS_* was obtained under conditions similar to optimal parameters of pectin extraction, at temperatures of 112.2 to 121.5 °C and times of 32.1 to 20 min, respectively, with a predicted optimum *Y_NS_* value of 82.6 g kg^−1^ (± 0.72) at 116.2 °C and 26.4 min, in very good agreement with experimental results (R^2^ = 0.976). The experimental validation data are described in Supplementary Materials (Tables S7A–S10A). 

In the hydrolysate liquor obtained in the optimal experimental region, pentoses (arabinose and xylose), as the main products of hydrothermolysis of hemicellulose, were found as largely dominant components (approx. 80 wt %). Therefore, it can be stated, that neutral monosaccharides were products of partial depolymerization of both extracted pectin and hemicellulose, which is a thermally unstable component of SBP and susceptible to hydrolysis [26]. 

Thus, after isolation of pectin from the extraction mixture and removal of alcohol, the recovery of neutral monosaccharides might also be possible. Regarding the experimental results, it can be concluded that SBP can be considered as suitable feedstock for neutral monosaccharides generation as a first step toward their further chemical and/or fermentative conversion to platform chemicals and biofuels.

## 3. Materials and Methods 

### 3.1. Materials and Chemicals

For all experiments, the SBP used was obtained from sugar beet harvested in 2016 on farms in Lower Silesian Province (Poland). Fresh SBP was obtained from Südzucker Polska S.A., Cukrownia Świdnica Trade (Poland) and stored at −18 °C. Before hydrothermal extraction, SBP was first washed with water and boiling ethanol [5,27]. After purification, SBP was oven-dried to constant mass at a temperature of 60 °C and ground to a grain size of below 1 mm. 

All chemicals were used without further purification. Monosaccharides (arabinose, galactose, glucose, mannose, rhamnose, and xylose) were purchased from Fluka. Water and uronic acids (galacturonic acid monohydrate and glucuronic acid) were derived from Sigma-Aldrich. Methanol, ethanol, isopropyl alcohol, and other reagents used in analytical determinations were purchased from POCh (Poland). All solvents and reagents were of analytical or HPLC grade, depending on the requirements of the analytical methods applied, and were used without further purification.

### 3.2. Reactor and Experimental Procedure

The hydrothermal extraction of pectin from SBP was performed in a 4576A-type batch reactor (Parr Instrument Company, Moline, IL, USA). 

Twenty grams of SBP and 180 g of water were used in each run. Preliminary experimental results of hydrothermal extraction of pectin from SBP (data not shown) allowed to set the initial extraction parameters (reaction temperature and time). A series of designed experiments was performed, in which extraction temperature and time were varied simultaneously, that is, pectin hydrothermal extraction from SBP was performed at temperatures ranging from 100 to 140 °C and durations from 0 (extraction was stopped once the intended temperature was reached) to 30 min. Before its use, HPLC-grade water was degassed in an ultrasonic bath and purged with nitrogen. The SBP suspension in water was introduced into the reaction vessel that had been originally preheated to approximately 60 °C. After the reactor was closed, the vessel containing the extraction mixture was purged a few times with nitrogen at 2 MPa and heated up at a rate of 6–10 °C min^−1^ to a predetermined temperature within 5 to 15 min (the time necessary to reach the final temperature) and kept at the same temperature with an accuracy of ±1 °C. When the desired temperature inside the reactor vessel was reached, the holding time started to be counted. Pectin hydrothermal extraction from SBP was either stopped after reaching the desired temperature (in this case the extraction time was considered as 0 min) or the suspension was maintained at the programmed temperature. The extraction was performed at pressures corresponding to or slightly exceeding the vapour pressure at a given temperature in all experimental runs. At the end of the programmed holding time, the vessel was cooled to approximately 60 °C within 5–10 min and, after the system was expanded, the vessel was emptied and rinsed with water, to reach the final volume of the water-soluble product fraction of 250 mL [28]. 

### 3.3. Separation of Products of Pectin Hydrothermal Extraction from Sugar Beet Pulp 

As a result of the hydrothermal extraction of pectin from SBP, a suspension containing water-soluble products (WS) was obtained. The WS fraction was separated from the solid post-extraction residue by vacuum filtration using PTFE membrane filters (Sartorius, SRP 15, 0.45 μm). 

The separated WS fractions were treated according to Ref. [9]. Pectin precipitates were recovered by centrifugation at 10,000× *g* for 15 min, washed with ethanol [22], and oven-dried to constant mass at a temperature of 40 °C. 

The yields (*Y_i_*) of all studied treatment products were calculated using the following equation (Equation (3)):

*Y_i_* (g kg^−1^) = *m_i_*/*m_SBP_* (3)

where *m_i_* is the mass (g) of product i, and *m_SBP_* is the mass (kg) of SBP subjected to hydrothermal treatment. 

In contrast, the yield (*Y_j_*) of components contained in the pectin extract was calculated using the following equation (Equation (4)):

*Y_j_* (g kg^−1^) = *m_j_*/*m_P_* (4)

where *m_j_* is the mass (g) of component j contained in the pectin, and *m_P_* is the mass (kg) of pectin. 

### 3.4. Analyses and Analytical Methods

The composition of SBP was determined using the typical analytical methods for biomass [29,30,31,32]. Pectin content in SBP was determined using the extraction method (pH 1.5, 4 h, 90 °C) applied in the work of Ref. [7]. The analytical determinations were performed in triplicate and the mean values were calculated.

The content of uronic acids, monosaccharides, and methoxyl and acetyl groups in SBP and pectin extract was determined after sulfuric acid hydrolysis [5] by high pressure liquid chromatography (HPLC). The methoxyl and acetyl groups were determined also by HPLC according to Voragen et al. [33]. 

The content of uronic acids, monosaccharides, carboxylic acids, and furfurals in the WS fractions was determined without acid hydrolysis by the HPLC method. The chromatographic method was performed on a Dionex Ultimate 3000 (System Thermo Scientific, Sunnyvale, CA, USA), consisting of a pump (LPG-3400SD), autosampler (WPS-3000TSL), and column oven (TCC-3000SD). The components were determined at a temperature of 60 °C in a Rezex ROA-Organic Acid H^+^ column (300 mm × 7.8 mm i.d., 8% cross-linked H^+^, Phenomenex, Torrance, CA, USA), equipped with a precolumn; 0.005 M sulfuric acid flowing at a rate of 0.6 mL min^−1^ was used as the mobile phase. Prior to use, the mobile phase was filtered through a 0.45 mm Millipore membrane filter. An ERC RefractoMax 520 (DataApex, Prague, Czech Republic) refractometric detector was employed to detect analytes. All analytical determinations were performed in duplicate and mean values were calculated. 

Weight-average molar weight of the pectin-enriched products was determined by high-performance size exclusion chromatography (HPSEC) on a Knauer HPLC system, equipped with a SEC s-4000 column (300 mm × 7.8 mm i.d.), Phenomenex, Torrance, CA, USA. Pectin solutions (0.2% *w*/*v*) were prepared by dissolving 100 mg of pectins in 50 mL sodium phosphate buffer with 0.04% sodium azide (NaN_3_) (pH 6.8) and clarified by centrifugation and filtration through PTFE membrane filters (Sartorius, Göttingen, Germany, SRP 15, 0.45 μm). Elution was carried out with 20 mM sodium phosphate buffer at pH 6.8 (with 0.04% sodium azide (NaN_3_)) at a flow rate of 0.5 mL min^−1^. Detection vas performed with a refractive index detector at ambient temperature. Molecular weight was calculated based on a comparison of elution times with those obtained for a pullulan standard kit (molecular weight range from 6.1 to 642 kDa, Shodex, Tokyo, Japan). 

The qualitative characteristics of pectin were determined by analyzing the attenuated total reflectance Fourier transformed infrared spectra (ATR-FTIR). The spectra of pectin extracts were obtained on the Nicolet iS50 FT-IR spectrometer system (Thermo Scientific Inc., Madison, WI, USA) with one bounce reflectance diamond ATR crystal accessory, coupled to a personal computer with Omnic analysis software. The ATR-FTIR spectra were recorded at room temperature at a 4 cm^−1^ resolution by averaging 32 scans in the spectral range of 400–4000 cm^−1^.

### 3.5. Modelling and Optimization Method

The OED methodology was used to determine process parameters ensuring an optimal yield of pectin and neutral monosaccharides. A detailed description of this methodology was published elsewhere [34,35]. From among the possible experimental matrices based on a quadratic model, the Doehlert array was selected [28,35]. The Doehlert matrices as experimental designs present the following advantages: uniform distribution of the experimental points in the chosen experimental domain, rotatable designs (constant variance at equal distance from the central point of the design), possibility to investigate adjacent or close experimental domains in a stepwise procedure, possibility to add new variables (although we did not use this feature in our work), number of required experiments smaller than that in other designs while achieving a similar precision of the estimates of the coefficients of the quadratic model.

The NEMROD software [36] was used for calculating (by the least-squares regression method) the coefficients of the model, evaluating the significance of the regression, and validity tests.

## 4. Conclusions

It was found that subcritical water technology is a promising step toward increasing the value of sugar beet pulp as a raw material for conversion to bioproducts.

Based on the obtained results, the yield of pectin extract reached highest values at temperatures of 112.6–124.9 °C and times of 26.2–15.8 min, respectively, and the maximum value was 121.1 g kg^−1^ at 118.1 °C and 21.5 min. The region of highest yield of neutral monosaccharides was found at ranges of temperatures from 112.2 to 121.5 °C and times from 32.1 to 20 min, respectively, with the estimated maximum of 82.6 g kg^−1^ at 116.2 °C and 26.4 min. In all experiments, two fractions of pectin were obtained: the first contained high molecular weight pectin (more than 642 kDa) and the second consisted of pectin with particles of small size (less than 6 kDa).

We propose a one-stage hydrothermal extraction of sugar beet pulp leading to effective co-production of pectin and neutral monosaccharides with a relatively high yield and satisfactory purity without the presence of an acidic catalyst. This might be an alternative route for increasing the value of this by-product and its use to produce fine chemicals that may be useful raw materials in food, pharmaceutical, polymer, agrochemical, and cosmetic industries. The obtained results are suitable for industrial upscaling and may provide an incentive to implement a new, environmentally friendly, simple, and effective method for the treatment of waste product of the sugar refining industry, which has been onerous to date.

Additional work is required to convert the remaining solid residue of the hydrothermal treatment of sugar beet pulp, still containing hemicellulose and rich in unreacted cellulose, lignin, and protein. 

## Figures and Tables

**Table 1:** Chemical composition of sugar beet pulp (SBP).

Component	Content (g kg^−1^)
Dry matter	210.1 ± 1.2*
Ash	23.7 ± 0.4
Total protein (N∙6.25)	88.4 ± 0.5
Diethyl ether extractable substance	55.2 ± 0.5
Pectin	134.8 ± 0.8
Hemicellulose	272.3 ± 2.4
Cellulose	140.0 ± 2.3
Lignin	88.6 ± 3.4
Remaining sucrose as saccharide after inversion	11.2 ± 1.2
Monosaccharides **:	
Arabinose	190.4 ± 0.5
Xylose	16.6 ± 1.0
Glucose	189.3 ± 0.9
Sum of galactose, mannose and rhamnose	80.1 ± 2.8
Uronic acids	188.3 ± 1.3
Acetic acid	1.7 ± 0.9
Methanol	4.2 ± 0.9

* Standard deviation. ** Determined in liquid fraction obtained after sulfuric acid hydrolysis of SBP.

**Table 2:** Doehlert matrices for experimental series: normalized (x_i_) and effective (u_i_) variables, and values of corresponding experimental and calculated responses, i.e., pectin yield (*Y_P_*) and sum of neutral monosaccharides yield (*Y_NS_*).

			Pectin				Sum of Neutral Monosaccharides	
Experiment	*x*_1_	*x*_2_	*u*_1_ *T* (°C)	*u*_2_ *t* (min)	*Y_P_-exp.* ** (g kg^−1^)	*Y_P_-cal.* *** (g kg^−1^)	*Y_NS_-exp.* ** (g kg^−1^)	*Y_NS_-cal.* *** (g kg^−1^)
1	+1	0	140	15.0	88.1	93.0	52.3	49.3
2	−1	0	100	15.0	91.1	86.2	38.7	41.8
3	+0.5	+0.866	130	30.0	102.5	97.6	61.1	64.2
4	−0.5	−0.866	110	0.0	57.3	62.2	38.9	35.8
5	+0.5	−0.866	130	0.0	89.4	84.5	55.9	59.0
6	−0.5	+0.866	110	30.0	108.9	113.2	83.0	79.9
7	0	0	120	15.0	118.9	118.0	78.9	78.4
7’ ^*^	0	0	120	15.0	117.9	118.0	77.0	78.4
7’’ ^*^	0	0	120	15.0	117.2	118.0	79.2	78.4

* Experiments repeated at the center of the experimental region to calculate the standard deviation of the response *Y_P_*: *s_P_* = 0.854; *Y_NS_*: *s_NS_* = 1.202. ** Experimental values. *** Calculated values.

**Table 3:** Chemical composition, degree of methylation, and degree of acetylation of pectin obtained in hydrothermal extraction of SBP.

Experiment	Uronic Acids (g kg^−1^)	Arabinose (g kg^−1^)	Glucose (g kg^−1^)	* (g kg^−1^)	Xylose (g kg^−1^)	DM ** (% mol)	DA *** (% mol)
1	499.2	131.6	11.5	181.3	12.5	31.9	16.1
2	506.1	119.6	9.4	152.9	11.1	54.2	18.0
3	499.0	133.0	13.4	171.9	15.1	39.2	13.6
4	503.9	114.4	9.5	118.1	12.4	53.2	21.6
5	504.2	121.6	9.5	157.2	13.7	45.0	21.3
6	499.9	121.9	9.3	160.6	13.9	47.2	13.8
7	511.4	122.4	9.4	165.1	13.4	51.9	16.9
7’	513.4	123.1	9.7	165.9	12.9	52.0	16.8
7’’	513.7	122.9	9.9	165.1	12.2	51.7	16.4

* Sum of galactose + mannose + rhamnose. ** Degree of methoxylation. *** Degree of acetylation.

## Supplementary Materials

The following are available online: Table S1A. Standard deviations of the coefficients of the quadratic models calculated for the pectin yield (*YP*) (Equation (3)) and for the sum of neutral monosaccharides yield (*Y_NS_*) (Equation (4)); Experimental validation - pectin yield (*Y*): Table S2A. Analysis of variance: response Y; Table S3A. Estimates of the coefficients: response Y; Table S4A. Statistics on the coefficients: response Y; Table S5A. Residuals: response Y; Table S6A. Material balances for hydrothermal extraction of pectin from 100.0 g of SBP; Experimental validation - neutral monosaccharides yield (Y): Table S7A. Analysis of variance: response Y; Table S8A. Estimates of and statistics on the coefficients: response Y; Table S9A. Statistics on the coefficients: response Y; Table S10A. Residuals: response Y.
