# Supplementary material for: Pectin and Neutral Monosaccharides Production during the Simultaneous Hydrothermal Extraction of Waste Biomass from Refining of Sugar—Optimization with the Use of Doehlert Design
Source: Molecules. 2019 Jan 29;24(3):472. doi: 10.3390/molecules24030472 (PMC6385035; doi:10.3390/molecules24030472)
Supplement: Supplementary file 1 [file molecules-24-00472-s001.pdf]

Pectin and neutral monosaccharides production during the simultaneous hydrothermal extraction of waste biomass from refining of sugar - optimization with the use of Doehlert design

Hanna Pińkowska [1], Małgorzata Krzywonos [2]*, Paweł Wolak [1] and Adrianna Złocińska [3]

Supplementary materials

Table 1A. Standard deviations of the coefficients of the quadratic models calculated for the pectin yield (Y_P) (Equation 1) and for the sum of neutral monosaccharides yield (Y_{NS}) (Equation 2).

	Y_P	Y_{NS}
	**Standard deviations**	
b_0	0.49	0.69
b_1	0.49	0.69
b_2	0.49	0.69
b_{11}	0.78	1.10
b_{22}	0.78	1.10
b_{12}	0.99	1.39

Experimental validation - pectin yield (Y)

Table 2A. Analysis of variance: response Y

Source of variation	Sum of squares	Degrees of freedom	Mean square	Ratio	Signif
Regression	3.05093E+0003	5	6.10187E+0002	835.8724	0.102 **
Residuals	1.44249E+0002	3	4.80829E+0001		
Validity	1.42789E+0002	1	1.42789E+0002	195.6011	0.330**
Error	1.46000E+0000	2	7.30000E-0001		
Total	3.19518E+0003	8			

Table 3A. Estimates of the coefficients : response Y

Standard deviation of the response	0.854
R2	0.955
R2A	0.880
R2 pred	N.D.
PRESS	5143.682
Number of degrees of freedom	2

*Corresponding author

Table 4A. Statistics on the coefficients : response Y

Name	Coefficient	F. Inflation	Standard deviation	t. exp.	Signif.
b0	118.000		0.493	239.21	< 0.01***
b1	3.393	1.00	0.493	6.88	1.70*
b2	18.505	1.00	0.493	37.51	0.0479***
b11	-28.395	1.04	0.780	-36.41	0.0503***
b22	-28.703	1.04	0.780	-36.80	0.0495***
b12	-21.882	1.00	0.987	-22.18	0.124**

Table 5A. Residuals: response Y

Run	$Y_{exp.}$	$Y_{calc.}$	Difference	Normalized	dU	Student-R	R-Student	D-Cook
1	88.120	92.998	-4.878	-5.710	0.833	-13.986	-13.986	163.001
2	91.090	86.212	4.878	5.710	0.833	13.986	13.986	163.001
3	102.500	97.622	4.878	5.710	0.833	13.986	13.986	163.001
4	57.300	62.178	-4.878	-5.710	0.833	-13.986	-13.986	163.001
5	89.400	84.522	4.878	5.710	0.833	13.986	13.986	163.001
6	108.300	113.178	-4.878	-5.710	0.833	-13.986	-13.986	163.001
7	118.900	118.000	0.900	1.053	0.333	1.290	0.130	0.139
8	117.900	118.000	-0.100	-0.117	0.333	-0.143	-0.014	0.002
9	117.200	118.000	-0.800	-0.936	0.333	-1.147	-0.116	0.110

Table 6A. Material balances for hydrothermal extraction of pectin from 100.0 g of SBP.

	Experiment						
	1	2	3	4	5	6	7*
Material out (g):							
WS fractions, including:	31.9	20.2	45.3	15.9	25.7	31.0	26.9
Pectin	8.8	9.1	10.3	5.7	8.9	10.9	11.8
Uronic acids	0.7	0.3	0.7	0.3	0.4	0.8	0.5
Monosaccharides	5.2	3.9	6.1	3.9	5.6	8.3	7.9
Carboxylic acids	2.0	1.2	2.3	1.1	1.6	2.8	2.0
Furfurals	2.0	0.0	2.0	0.0	0.0	0.0	0.0
Protein	3.3	1.8	1.5	0.8	0.7	0.4	0.8
Unidentified products in the WS fractions**	9.9	3.9	22.4	4.1	8.5	7.8	3.9
% Recovery of products in the WS fractions***	27.6	45.0	22.7	35.9	34.6	35.2	43.9
WI fractions (g)	45.1	79.6	51.3	83.7	72.5	68.6	72.6
Losses**** + gas fractions (g)	23.0	0.2	3.4	0.4	1.8	0.4	0.5

*- mean value of 3 repeated experiments, **- losses (L) calculated according to the equation L = mass of WS fractions minus mass of pectin, uronic acids, monosaccharides, carboxylic acids, furfurals and protein present in the WS fractions, ***- % recovery was calculated in proportion to content of pectin in the WS fractions obtained in performed experimental series, ****- losses in all obtained product fractions were calculated according to equation L = 100 g minus mass of WS fraction minus mass of WI fraction.

Experimental validation - neutral monosaccharides yield (Y)

Table 7A. Analysis of variance: response Y

Source of variation	Sum of squares	Degrees of freedom	Mean square	Ratio	Signif
Regression	2.38955E+0003	5	4.77910E+0002	330.6347	0.248**
Residuals	5.98093E+0001	3	1.99264E+0001		
Validity	5.69184E+0001	1	5.69194E+0001	39.3781	2.100**
Error	2.89087E+0000	2	1.44543E+0000		
Total	2.44936E+0003	8			

Table 8A. Estimates of and statistics on the coefficients : response Y

Standard deviation of the response	1.202
R2	0.976
R2A	0.935
R2 pred	0.161
PRESS	2055.567
Number of degrees of freedom	2

Table 9A. Statistics on the coefficients : response Y

Name	Coefficient	F. Inflation	Standard deviation	t. exp.	Signif.
b0	78.377		0.694	112.91	0.0123***
b1	3.720	1.00	0.694	5.36	2.98*
b2	14.244	1.00	0.694	20.52	0.145***
b11	-32.837	1.04	1.098	-29.92	0.0703***
b22	-13.944	1.04	1.098	-12.70	0.0411***
b12	-22.436	1.00	1.388	-16.16	0.240**

Table 10A. Residuals: response Y

Run	$Y_{exp.}$	$Y_{calc.}$	Difference	Normalized	dU	Student-R	R-Student	D-Cook
1	52.340	49.260	3.080	2.562	0.833	6.275	6.275	32.815
2	38.740	41.820	-3.080	-2.562	0.833	-6.275	-6.275	32.815
3	61.110	64.190	-3.080	-2.562	0.833	-6.275	-6.275	32.815
4	38.880	35.800	3.080	2.562	0.833	6.275	6.275	32.815
5	55.870	58.950	-3.080	-2.562	0.833	-6.275	-6.275	32.815
6	82.980	79.900	3.080	2.562	0.833	6.275	6.275	32.815
7	78.910	78.377	0.533	0.444	0.333	0.543	0.120	0.025
8	77.000	78.377	-1.377	-1.145	0.333	-1.402	-0.316	0.164
9	79.220	78.377	0.843	0.701	0.333	0.859	0.191	0.062
